# Low vision: we can all do more

**Published:** 2012

**Authors:** Hasan Minto, Clare Gilbert

**Affiliations:** Regional Director for the Eastern Mediterranean, International Centre for Eyecare Education.; Co-director, International Centre for Eye Health, London School of Hygiene and Tropical Medicine, Keppel Street, London WC1E 7HT, UK; Clinical Advisor, Sightsavers.

**Figure:**
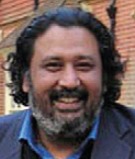
Hasan Minto

**Figure:**
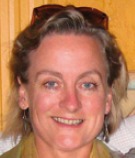
Clare Gilbert

Despite all the treatments, operations, and medication at our disposal, there is still a significant number of people whose sight we cannot fully restore.

What happens to these patients once they leave our care? Without the necessary support, advice, and low vision devices, their remaining vision will not be very good; this can make life a struggle.

Support may be difficult to find, as low vision services are often inadequate or inaccessible in many low- and middle-income countries. Professionals, such as rehabilitation workers, ophthalmologists, mid-level eye care workers, optometrists/refractionists, and special education teachers, may not know what to do about people with low vision, leaving them with no-one else to turn to.

Individuals who can only see light or movement of large objects will need rehabilitation that focuses on non-visual strategies for learning and daily tasks. However, there are many people who have slightly better vision, but are still classified as blind, who have the potential to use their sight. These people could benefit from low vision care, which may include refraction, provision of magnifiers, and/or environmental modifications.

The World Health Organization defines a person who needs to be assessed for low vision care as someone “who has impairment of visual functioning even after treatment and/or standard refractive correction, and has a visual acuity of less than 6/18 down to and including light perception, or a visual field of less than 10 degrees from the point of fixation, but who uses, or is potentially able to use, vision for the planning and/or execution of task.”

The important part of this definition is that people should only be assessed for low vision interventions once all other treatments the person needs (surgical, medical and/or optical) have been given. The definition also emphasises the importance of vision for day-to-day functioning.

**Figure:**
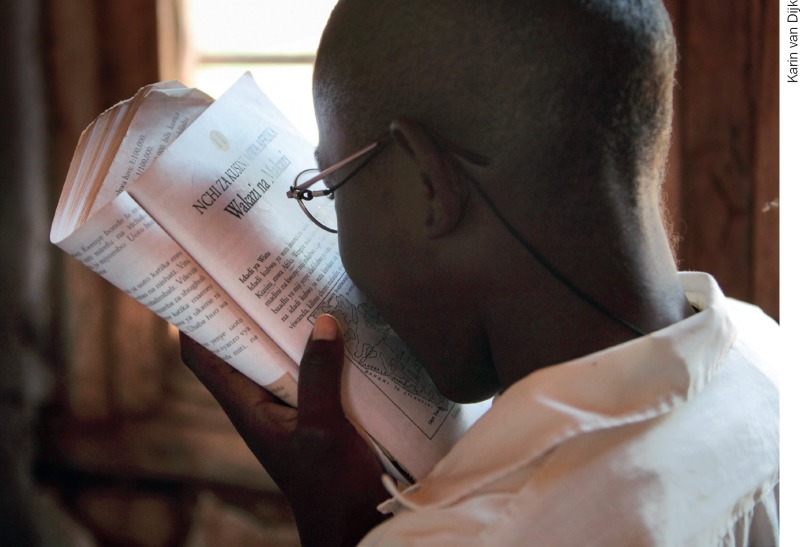
Use real tasks to assess near vision. TANZANIA

People who may be able to benefit from low vision care will want to do a range of different things. In many low- and middle-income countries, for example, many people with low vision are aged over 50 years and cannot read or write. They will have different needs, and require different services, compared to children or adults in employment.

Low vision has a significant impact on people's lives. People with low vision may struggle to look after themselves without help. Having low vision affects their status in the eyes of others and can make social situations difficult. It reduces the ability of people to pursue an education, to look after their children, and to earn an income. People with low vision are also at greater risk of falls and death.

With our support, people with low vision can make better use of their sight to do the things they want and need to do. We hope this issue will show you how.

